# Activity of *Ocimum basilicum*, *Ocimum canum*, and *Cymbopogon citratus* essential oils against *Plasmodium falciparum* and mature-stage larvae of *Anopheles funestus* s.s.

**DOI:** 10.1051/parasite/2014033

**Published:** 2014-07-07

**Authors:** Patrick Akono Ntonga, Nicolas Baldovini, Elisabeth Mouray, Lengo Mambu, Philippe Belong, Philippe Grellier

**Affiliations:** 1 Laboratory of Animal Biology, Department of Animal Biology, Faculty of Science, University of Douala P.O. Box 24157 Douala Cameroon; 2 Institut de Chimie de Nice UMR 7272, Faculté des Sciences, University of Nice Sophia Antipolis, Parc Valrose 06108 Nice France; 3 Muséum National d’Histoire Naturelle, UMR 7245 CNRS, CP 52 61 rue Buffon 75231 Paris Cedex 05 France; 4 Université de Limoges, Laboratoire de Chimie des Substances Naturelles, EA 1069, Institut GEIST, Faculté de Pharmacie 2 rue Docteur Marcland 87025 Limoges Cedex France; 5 Higher Teacher Training College, University of Yaoundé I Yaoundé Cameroon

**Keywords:** *Plasmodium falciparum*, *Anopheles funestus* s.s., Essential oils, Biocides, Anti-malarial drugs

## Abstract

The biological activities of essential oils from three plants grown in Cameroon: *Ocimum basilicum*, *Ocimum canum*, and *Cymbopogon citratus* were tested against *Plasmodium falciparum* and mature-stage larvae of *Anopheles funestus.* Gas chromatography and gas chromatography – mass spectrometry analyses showed that the main compounds are geranial, 1,8-cineole and linalool in *C. citratus*, *O. canum* and *O. basilicum*, respectively. Larvicidal tests carried out according to the protocol recommended by the World Health Organization showed that the essential oil of leaves of *C. citratus* is the most active against larvae of *An. funestus* (LC_50_ values = 35.5 ppm and 34.6 ppm, respectively, for larval stages III and IV after 6 h of exposure). Besides, the in vitro anti-plasmodial activity evaluated by the radioisotopic method showed that the *C. citratus* oil is the most active against *P. falciparum*, with an IC_50_ value of 4.2 ± 0.5 μg/mL compared with *O. canum* (20.6 ± 3.4 μg/mL) and *O. basilicum* (21 ± 4.6 μg/mL). These essential oils can be recommended for the development of natural biocides for fighting the larvae of malaria vectors and for the isolation of natural products with anti-malarial activity.

## Abstract (fr)

Les activités biologiques des huiles essentielles de trois plantes cultivées au Cameroun, *Ocimum basilicum*, *Ocimum canum* et *Cymbopogon citratus* ont été testées contre *Plasmodium falciparum* et les stades mûrs des larves d’*Anopheles funestus* s.s. Les analyses par chromatographie en phase gazeuse et chromatographie en phase gazeuse–spectrométrie de masse ont montré que les principaux composés sont le géranial, le 1,8-cineole et le linalool, respectivement, dans les huiles essentielles de *C. citratus*, *O. canum* et *O. basilicum*. Les tests larvicides réalisés selon le protocole recommandé par l’Organisation Mondiale de la Santé ont montré que l’huile essentielle des feuilles de *C. citratus* est la plus active vis-à-vis des larves d’*An. funestus* s.s. (CL_50_ = 35,5 ppm et 34,6 ppm respectivement pour les larves de stades III et IV après 6 heures d’exposition). En outre, l’activité antiplasmodiale *in vitro* évaluée par la méthode radio-isotopique a montré que l’huile essentielle de *C. citratus* est la plus active contre *P. falciparum* avec une CL_50_ de 4,2 ± 0,5 μg/ml comparée à celles d’*O. canum* (20,6 ± 3,4 μg/ml) et d’*O. basilicum* (21 ± 4,6 μg/ml). Ces huiles essentielles peuvent être recommandées pour le développement de biocides naturels contre les larves des vecteurs du paludisme et pour l’isolement de molécules naturelles ayant une activité anti-plasmodiale.

## Introduction

Malaria remains one of the most deadly diseases in the world. About 154–289 million persons are infected each year with 490–836 thousand deaths, mostly in children under 5 years of age. About 90% of this burden is recorded in Africa [47]. Despite numerous efforts by the scientific community to reduce the prevalence of malaria to its lowest level, the desired result is far from being achieved. This is linked to the approximate application of preventive and curative measures, the poverty of populations, and the resistance of both *Plasmodium* and malaria vectors to anti-malarial drugs and insecticides, respectively. In Cameroon, several studies have demonstrated the resistance of vectors and *Plasmodium* to drugs [10, 15–17, 35]. This double resistance is a major obstacle to the prevention and treatment of malaria. Faced with this problem, the use of eco-friendly anti-plasmodial and insecticidal natural products is strongly encouraged. In the African tradition, the use of plants with insecticidal or anti-plasmodial properties is well known. Pyrethroids and tobacco have long been used as control agents against insects [12]. *Artemisia annua*, a plant traditionally used in Chinese medicine against fevers, is now the source of artemisinin, a drug recommended by the World Health Organization (WHO) for the treatment of malaria. Plants are thus turning out to be a potential source of new insecticides and antimalarial drugs for the future. In Cameroon, little work on the insecticidal efficacy and anti-plasmodial activity of essential oils of plants has been conducted [39, 40]. In this study, we extracted and analyzed the essential oils of three plants widely used in Cameroon as insect repellents: *Ocimum basilicum*, *Ocimum canum*, and *Cymbopogon citratus*, and we determined their biological activities on *Plasmodium falciparum* and the larvae of *Anopheles funestus* s.s.

## Materials and methods

The experiments comply with the current laws of Cameroon in which they were performed.

### Plant harvesting and extraction of essential oils

Plants were chosen for their traditional use as insect repellents. Indeed, in the villages of the rainforest where high malaria endemicity occurs, it is strongly recommended to place these plants in the four corners of the room to repel mosquitoes. Plant material was collected in June 2009 in an experimental field free from pesticide treatment of the city of Douala (Cameroon). Specimens collected were then identified at the National Herbarium in Yaoundé (Cameroon) and referenced under the following numbers: *Ocimum basilicum* (6899SRFcam); *Ocimum canum* (13497SRFcam); *Cymbopogon citratus* (48536/SRF). The fresh leaves (16 kg) of each plant specimen were washed with water, cut into small pieces, and subjected to hydrodistillation using a Clevenger-type apparatus for 5 h. The essential oil was collected by decantation, and was dried on anhydrous sodium sulfate. It was stored in dark glass bottles and kept at 4 °C before analysis. The extraction yields were 0.44, 0.11, and 0.60% for *O. canum*, *O. basilicum*, and *C. citratus*, respectively.

### GC and GC/MS analyses

Gas chromatography and gas chromatography – mass spectrometry (GC and GC-MS) analyses were carried out using an Agilent 6890N gas chromatograph apparatus equipped with a flame ionization detector (FID) and coupled to a quadrupole Agilent 5973 network mass selective detector working in electron impact mode at 70 eV (scanning over 35–350 amu range). The gas chromatograph was equipped with two HP-1 fused silica capillary columns (PDMS, 50 m × 0.2 mm i.d., film thickness: 0.33 μm). The analytical parameters (identical for GC and GC-MS analyses unless specified) were the following: the carrier gas was helium at a flow rate of 1 mL/min. The oven temperature was programmed from 60 to 250 °C at 2 °C/min and held isothermal for 40 min. The injector (split mode, ratio 1/100) temperature was 250 °C. The FID temperature was set at 250 °C, and in the GC-MS analyses, the temperatures of the ion source and transfer line were 170 and 280 °C, respectively. To remove some coelutions, another series of analysis were performed with the same analytical parameters as above, but with a HP-5 capillary column. In both cases, retention indices (RI) were determined from the retention times of a series of *n*-alkanes with linear interpolation. The constituents of the essential oil were identified by comparison of their mass spectral pattern and RI with those of pure compounds registered in commercial libraries and literature data and with a laboratory-made database built from authentic compounds. Quantitative data were obtained by internal standardization using Nonane as an internal standard. For a given compound, its relative response factor (RRF) was predicted according to its chemical class [11].

### Rearing of larvae of *Anopheles funestus* s.s.

Tests were carried out on a strain of *An. funestus* s.s. domesticated in the Medical Entomology Laboratory, University of Yaoundé I (Cameroon). The larvae were reared in the insectarium of that laboratory in plastic containers (20 × 10 × 10 cm). Larval density was 100 larvae for 1 L of spring water. The food used was TetraBaby fish food [13]. The average temperature inside the insectariums was kept constant by a continuously operating heater (28.2 °C ± 0.9 °C) with a relative humidity of 80%.

### Culture of *Plasmodium falciparum*

The chloroquine-resistant strain FcB1/Colombia of *P. falciparum* was maintained on human red blood cells in RPMI 1640 medium, containing 25 mM HEPES, pH 7.3, 2 g/L sodium bicarbonate, 2 g/L glucose, penicillin, and streptomycin [42]. The medium was enriched with 10% heat-inactivated human serum. The red blood cells and serum used came from the French Blood Establishment. Culture was performed at a hematocrit of 2% in flasks of 25 and 75 mL capacity and maintained under an oxygen-deficient atmosphere at 37 °C. The culture medium was renewed once a day. A Giemsa-stained blood smear was done daily to control parasitemia.

## Bioassays

### Larvicidal tests

Larvicidal tests were carried out according to the protocol recommended by the WHO [46]. Different stock solutions were prepared in ethanol from the crude essential oil of each plant to obtain after dilution in water the final concentrations of 250 ppm, 200 ppm, 150 ppm, 100 ppm, and 50 ppm. The tests were performed in 5-cm-diameter beakers, each containing 99 mL of spring water, 25 larvae of the same stage, and 1 mL of a diluted solution of essential oil. For each concentration of essential oil, the test was repeated five times to minimize errors. A beaker to which was added only 1 mL of 5% (v/v) ethanol solution in water constituted the control. This beaker was prepared under identical conditions to the other tested beakers. The counting of dead larvae was performed every 30 min for 6 h, after exposure to the different concentrations of oils. All larvae that became immobile after exposure to dilute solutions of essential oils were removed from the test medium and rinsed with water (devoid of any chlorinated substance), and were placed under observation for 24 h. It was at the end of the 24th hour that mortality was confirmed. At the end of the observation period, no immobile larva was returned to life.

### Anti-plasmodial test

The in vitro anti-plasmodial activity of essential oils was evaluated by the radioisotopic method [14]. This method determines the inhibition of parasite growth in culture in the presence of various concentrations of molecules by measuring the incorporation of [^3^H] hypoxanthine into parasite nucleic acids. Assays were performed in 96-well plates as previously described [22]. Briefly, essential oils were prepared in culture medium, serially diluted with culture medium and added to asynchronous parasite cultures (1% parasitemia, 1% final hematocrit, 200 μL final volume *per* well) for 24 h, at 37 °C, prior to the addition of 0.5 μCi of [^3^H] hypoxanthine (1–5 Ci/mmol; Amersham, Les Ulis, France) *per* well, for 24 h. Plates were incubated at 37 °C under a humid and oxygen-deficient atmosphere. Assays were interrupted by freezing plates at −80 °C. After thawing, the contents of wells were collected on glass fiber filters (Wallac^®^, USA) using a cell collector (Filter Harvester, USA). After addition of scintillation fluid (Perkin Elmer^®^, USA), the radioactivity (counts *per* minute) was measured using a spectrophotometer (1450-Microbeta Trilux, USA). The growth inhibition for each drug concentration was determined by comparison of the radioactivity incorporated in the treated culture with that in the control culture (without drugs) maintained on the same plate. The concentration causing 50% inhibition (IC_50_) was obtained from the drug concentration-response curve and the results were expressed as the mean ± the standard deviations determined from at least three independent experiments.

To avoid parasite growth inhibition due to a phenomenon of diffusion of essential oils from nearby wells, preliminary tests were conducted to determine the highest essential oil concentration for which such an inhibition was not measured. For that, the crude essential oil was only serially diluted with culture medium in one row of a plate, the other rows containing only culture medium. Parasites were added to all the wells of the plate and the plate was processed as described above. The highest concentration of essential oil showing no inhibition of parasite growth in the surrounding wells was used as a starting concentration to further determine the intrinsic anti-plasmodial activity of the essential oil.

### Statistical analysis

Analyses were performed by using the statistical software SPSS version 19.0. The Kruskal Wallis H test (*p* < 0.05) was used to compare the mean numbers of mortality of larvae. The Henry simplified table that transforms the percentages of larval mortality into probit was used to determine the lethal concentration required to kill 50% (LC_50_) of larvae.

## Results and discussion

### Chemical composition of essential oils

The chemical compositions of each essential oil are given in Table 1. In each case, the main components are monoterpenoids: geranial and neral in *Cymbopogon citratus*, and linalool and 1,8-cineole in both *Ocimum* species, which also contain ca. 8% of eugenol. Low amounts of some sesquiterpenes are also observed in *Ocimum* essential oils. The chemical composition of the essential oil of *O. basilicum* is in line with that described previously from plants collected in Cameroon [39] albeit the amount of limonene is lower. It should be noted that the same article reports the presence of methyl chavicol as the main constituent in a sample from Congo. This component often accompanies linalool [30, 33] and is sometimes the major constituent [3, 9, 29]. The same situation is observed with methyleugenol [1, 2, 34]. Thus, we were interested in evaluating the bioactivity of the sample described in our study since it was free of methylchavicol and its methyleugenol content was very low. Indeed, both of these compounds are known carcinogens [26, 31], and would obviously prevent any anti-malarial use of the essential oil. According to the literature, *Ocimum canum* essential oils show an impressive chemical variability, with compositions characterized by the predominance of various compounds: camphor [7], citral [6], terpinen-4-ol [36, 44], *α*-terpineol [8], and methyl trans-cinnamate [27] in addition to the 1,8-cineole- or linalool-rich chemotypes related to the sample described in this work [40, 41, 44]. In contrast, the composition of *Cymbopogon citratus* essential oil is much more homogeneous, always with geranial and neral as the main constituents whatever the origin of the plant. The samples characterized in Burkina Faso [28], Brazil and Portugal [18], and Benin [5] confirm this observation.

**Table 1.** Chemical composition of essential oils from leaves of *Cymbopogon citratus* (DC), *Ocimum canum* Sims and *Ocimum basilicum* L.

Compounds	ID	RI^a^	RI Litt^a^	Mass percentages (%)^b^		
				*C. citratus*	*O. canum*	*O. basilicum*
Hydrocarbon monoterpenes						
*α*-thujene	MS, RI	928	924	–	0.17	0.09
*α*-pinene	MS, RI	933	932	–	1.66	0.51
Camphene	MS, RI	945	946	–	0.27	
Sabinene	MS, RI	967	968	–	0.49	0.34
*β*-pinene	MS, RI	973	972	–	1.94	0.96
Myrcene	MS, RI	984	983	11.43	0.97	0.99
*α*-terpinene	MS, RI	1011	1009	–	0.32	0.18
*p*-cymene	MS, RI	1017	1014	–	0.95	0.34
Limonene	MS, RI	1023	1024	0.04	~3^c^	~1^c^
*β*-phellandrene	MS, RI	1026	1021	–	–	–
(*Z*)-*β*-ocimene	MS, RI	1028	1024	0.27	0.11	0.08
(*E*)-*β*-ocimene	MS, RI	1040	1037	–	2.44	1.18
*γ*-terpinene	MS, RI	1051	1051	0.22	0.70	0.38
Terpinolene	MS, RI	1081	1080	–	0.38	0.13
Perillene	MS	1088	1063	–	0.14	–
(*E*)-*β*-epoxyocimene	MS	1126	–	–	2.04	–
Oxygenated monoterpenes						
1,8-cineole	MS, RI	1027	1022	0.22	<29.04^c^	<13.95^c^
5-Isopropyl-2-methylbicyclo[3.1.0]hexan-2-ol	MS, RI	1056	1050	–	0.80	0.10
*cis*-linalool oxide (furanoid)	MS, RI	1058	1062			0.12
Fenchone	MS, RI	1072	1071	–	2.87	0.64
*trans*-linalool oxide (furanoid)	MS	1074	–	–	0.07	0.10
Linalool	MS, RI	1092	1086	0.72	19.07	51.86
Isocitral	MS	1123	–	0.30	–	–
Camphor	MS, RI	1124	1123	–	2.00	0.13
(*E*)-chrysanthenal	MS	1128	–	0.17	–	–
Citronellal	MS, RI	1132	1134	0.15	–	–
(*Z*)-chrysanthenol	MS, RI	1143	1153	1.17	–	–
*δ*-terpineol	MS	1151	–	–	0.45	0.23
(*E*)-chrysanthenol	MS	1161	–	1.62	–	–
Terpinen-4-ol	MS, RI	1168	1165	–	7.53	2.98
*α*-terpineol	MS, RI	1177	1175	–	2.31	1.32
Fenchyl acetate (endo)	MS, RI	1208	1200	–	0.12	0.11
Neral	MS, RI	1228	1216	30.21	–	0.64
Geraniol	MS, RI	1249	1238	8.19	–	0.18
Geranial	MS, RI	1259	1246	32.82	–	0.79
Isobornyl acetate	MS, RI	1269	1275	–	0.17	0.13
Neryl acetate	MS, RI	1359	1344	0.46	–	–
Hydrocarbon sesquiterpenes						
*α*-copaene	MS, RI	1376	1376	–	0.21	0.07
*β*-elemene	MS, RI	1388	1386	–	0.67	0.39
*β*-caryophyllene	MS, RI	1419	1420	0.05	0.42	0.4
*trans*-*α*-bergamotene	MS, RI	1434	1433	0.07	3.49	1.39
Aromadendrene	MS, RI	1436	1446	–	0.22	0.14
*α*-humulene	MS, RI	1453	1449	–	0.51	0.18
*epi*-bicyclosesquiphellandrene	MS	1459	–	–	0.17	0.13
Germacrene-D	MS, RI	1477	1476	–	0.76	0.49
Bicyclogermacrene	MS, RI	1492	1491	–	0.30	0.21
*δ*-guaiene	MS, RI	1500	1495	–	0.31	0.18
*γ*-cadinene	MS, RI	1508	1511	–	0.70	0.50
Calamenene	MS, RI	1511	1513	–	0.07	0.04
*δ*-cadinene	MS, RI	1514	1516	–	0.30	0.11
(*E*)-*α*-bisabolene	MS	1532	–	–	0.97	0.26
Oxygenated sesquiterpenes						
Spathulenol	MS, RI	1566	1567	–	0.12	0.08
Aromatics						
Eugenol	MS, RI	1334	1330	0.13	8.01	8.39
1,2-dimethoxy-4-propenylbenzene	MS, RI	1369	1373	–	0.24	0.09
Others						
(*Z*)-3-hexen-1-ol	MS, RI	834	839	–	0.17	0.24
6-methyl-hept-5-en-2-one	MS, RI	964	964	0.96	–	–
Oct-1-en-3-ol	MS, RI	965	964	–	0.15	0.26
(*Z*)-3-hexen-1-yl acetate	MS, RI	987	987	–	0.2	–
Octyl acetate	MS, RI	1193	1187	–	0.11	0.08
Undecan-2-one	MS, RI	1276	1274	0.17	–	–
Tridecan-2-one	MS, RI	1474	1478	0.10	–	–

a Retention index on HP-1 column.

b Percentage calculated by internal standardization. The following RRF were used for calculation: 1.03 (monoterpenes), 0.98 (sesquiterpenes), 1.30 (alcohols, ketones, and aldehydes), 1.59 (esters), 0.99 (aromatic hydrocarbons), and 1.28 (ethers), with nonane as an internal standard.

c On both columns, limonene and 1,8-cineole are coeluted; however, the latter is the main component with a proportion of limonene estimated to be inferior to 1/10 of that of 1,8-cineole.

### Biological activities of essential oils

**Figure 1.**
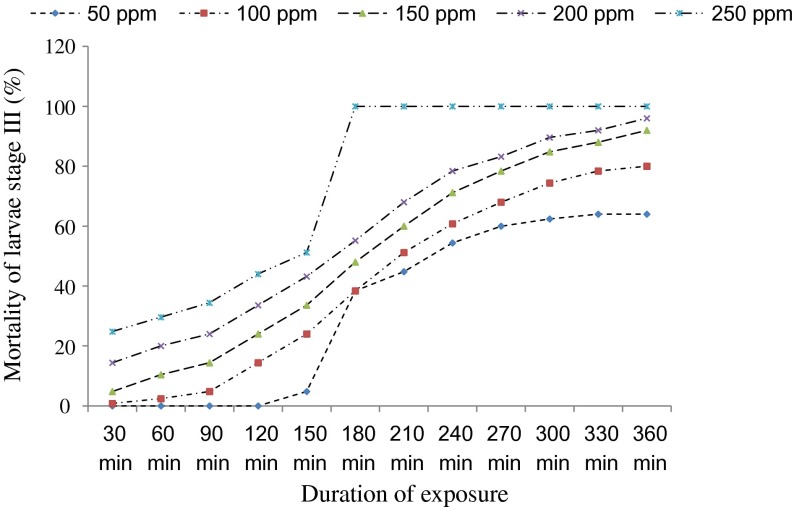
Percentage of mortality of stage III larvae of *Anopheles funestus* s.s. depending on the duration of exposure to different concentrations of the essential oil of *Cymbopogon citratus*.

**Figure 2.**
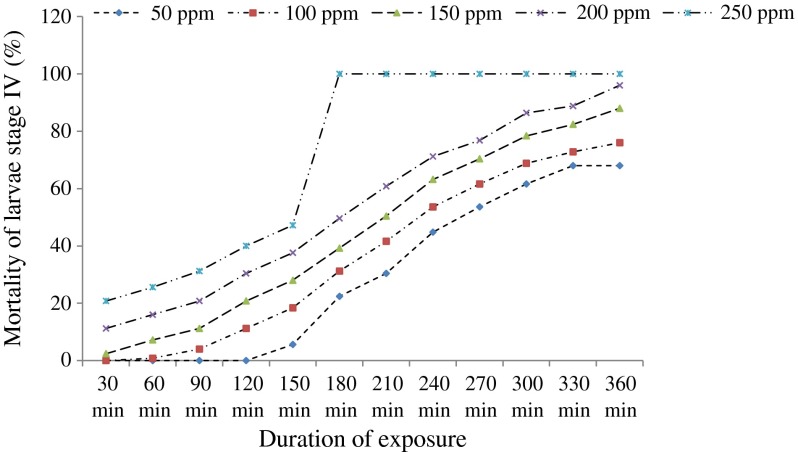
Percentage of mortality of stage IV larvae of *Anopheles funestus* s.s. depending on the duration of exposure to different concentrations of the essential oil of *Cymbopogon citratus*.

The Kruskal-Wallis H test shows that the mortality of *An. funestus* s.s. larvae varies according to the concentrations of essential oils and their botanical origin (Table 2). The mortality also varies according to the duration of exposure of larvae (Figs. 1 and 2). Essential oils of the three plants were active on the older larval stages of *An. funestus* s.s., but at different levels of toxicity. The essential oil of *C. citratus* leaves is the most active and the only one that caused total mortality of the larval stages III and IV at 250 ppm concentration after 6 h of exposure. At equal concentrations, oils of *O. canum* and *O. basilicum* leaves induced a partial mortality of larval stages III and IV after 6 h of exposure (Table 2).

**Table 2.** Mortality of larvae of *Anopheles funestus* s.s. depending on the concentration and botanical origin of essential oils studied after 6 h of exposure (H Kruskal-Wallis test, *P* < 0.05).

	Essential oils	Concentrations (ppm)						*P*
		250	200	150	100	50	Control	
Mean numbers of dead stage IV larvae	*Cymbopogon citratus*	25 ± 0.0	24 ± 1.4	22 ± 1.0	19 ± 0.7	17 ± 0.0	0 ± 0.0	0.002
	*Ocimum canum*	21 ± 1.2	16 ± 1.0	14 ± 0.7	12 ± 0.7	10 ± 1.0	0 ± 0.0	0.005
	*Ocimum basilicum*	20 ± 0.7	15 ± 1.2	12 ± 1.4	7 ± 1.2	4 ± 0.7	0 ± 0.0	0.01
	*P*	0.0001	0.0001	0.0002	0.0001	0.0001	–	–
Mean numbers of dead stage III larvae	*Cymbopogon citratus*	25 ± 0.0	24 ± 1.4	23 ± 1.5	20 ± 1.7	16 ± 0.7	0 ± 0.0	0.001
	*Ocimum canum*	22 ± 1.4	19 ± 0.7	13 ± 1.0	13 ± 0.7	12 ± 1.2	0 ± 0.0	0.002
	*Ocimum basilicum*	21 ± 1.8	16 ± 0.7	13 ± 1.4	9 ± 1.2	3 ± 0.7	0 ± 0.0	0.01
	*P*	0.0001	0.0002	0.0001	0.0002	0.0001	–	–

Average of five tests each covering 25 larvae. *P* = probability.

LC_50_ values (Tables 3 and 4) were determined after 6 h of exposure from the simplified table of the transformation into probit of the cumulative frequencies of Henry [20]. They permitted us to classify oils according to their degree of toxicity. The essential oil of *Cymbopogon citratus* is the most active, followed by those of *O. canum* and *O. basilicum* with LC_50_ values for larval stage IV of 34.6 ppm, 91.2 ppm, and 144.5 ppm, respectively, and 35.5 ppm, 74.1 ppm, and 131.8 ppm for larval stage III. It is also worth noting the speed of action of the essential oil of *Cymbopogon citratus* against the larvae of *An. funestus* s.s. (100% larval mortality was recorded within 3 h of exposure; Figs. 1 and 2) compared with conventional larvicides such as spinosad (100% larval mortality of *Aedes aegypti* was recorded within 24 h of exposure) [4].

**Table 3.** Larvicidal activity of essential oils on the fourth-stage larvae of *Anopheles funestus* s.s.

Essential oils	*LC_50_ (ppm)
*Ocimum basilicum*	144.5
*Ocimum canum*	91.2
*Cymbopogon citratus*	34.6

* Concentration causing 50% mortality of larvae.

**Table 4.** Larvicidal activity of essential oils on the third-stage larvae of *Anopheles funestus* s.s.

Essential oils	*LC_50_ (ppm)
*Ocimum basilicum*	131.8
*Ocimum canum*	74.1
*Cymbopogon citratus*	35.5

* Concentration causing 50% mortality of larvae.

Essential oils of the three plants were active in vitro on *P. falciparum* (Table 5). The intrinsic anti-plasmodial activity of each essential oil was evaluated for a range of concentrations for which we previously verified the absence of an inhibitory effect of one well to another due to the volatile inhibitory activity of the chemicals. Under these conditions, the essential oil of *C. citratus* appeared to be the most active, with an IC_50_ value of 4.2 ± 0.5 μg/mL, five times lower than those of *O. canum* and *O. basilicum.*

**Table 5.** Anti-plasmodial activity of essential oils *on Plasmodium falciparum*.

Essential oils	*IC_50_ ± *SD* (μg/mL)
*Ocimum basilicum*	21.0 ± 4.6
*Ocimum canum*	20.6 ± 3.4
*Cymbopogon citratus*	4.2 ± 0.5

* Concentration inhibiting 50% of parasite growth. Mean and standard deviation (*SD*) were determined from at least three independent experiments.

This efficiency of *C. citratus* could be due to the presence of a large proportion of citral (neral, 30.21% and geranial, 32.82%) in its chemical composition. The biological activity of these compounds was previously mentioned in the in vivo evaluation of lemongrass essential oil against *Plasmodium berghei* [39] and in the evaluation of in vitro antifungal activity of essential oils of citrus on the mycelial growth of *Phaeoramularia angolensis* [24]. In *P. falciparum*, isoprenoid biosynthesis is essential for parasite growth and depends on the DOXP/2-C-methyl-D-erythritol-4-phosphate (MEP) pathway, in contrast to humans, where isoprenoids are synthesized via the mevalonate pathway [25]. The *P. falciparum* MEP pathway constitutes an attractive target for the development of new anti-malarials, as evidenced by the in vivo anti-malarial activity of fosmidomycin, an inhibitor of DOXP reductoisomerase [45]. By their richness in monoterpenes, molecules with a similar chemical structure to the intermediates of the isoprenoid pathways, these essential oils may inhibit *P. falciparum* growth by acting upon the isoprenoid biosynthesis, as demonstrated for the monoterpene linalool [21], a major compound of the essential oils of *O. canum* and *O. basilicum* (Table 1). It is also possible to attribute at least partially the origin of the efficiency of *C. citratus* against the larvae of *An. funestus* s.s. to citral; because its toxic effect in the evaluation of the larvicidal activity of the essential oil of *C. citratus* on larvae of *Anopheles gambiae* was demonstrated [40]. Recently, Freitas et al. [19] also attributed the larvicidal and insecticidal activities of *C. citratus* against *Ae. aegypti* to citral. However, it would be unwise to believe that the toxicity of a plant is necessarily linked to the nature of the dominant compound. Several compounds acting in synergy can also be the source of the toxic effectiveness of an essential oil [32]. Moreover, the larvicidal activity of volatile oils of *O. canum* and *O. basilicum* is probably due to their richness in phenolic compounds. The efficacy of these compounds was previously demonstrated on fungi such as *Alternaria alternata* and *Colletotrichum capsicii* [43]. Phenolic compounds are also known to have ovicidal and larvicidal properties against various insect species [23]. They enhance their toxic potential when associated with terpinene [38]. The association terpinene/phenolic compounds is at the origin of the effectiveness of essential oils of *O. canum* and *O. basilicum*. These compounds lead to an inhibition of growth regulators in insects [37].

The present study showed that the essential oils from the leaves of *O. basilicum*, *O. canum*, and *C. citratus* from Cameroon have interesting insecticidal and anti-plasmodial properties. Their effectiveness in inhibiting the growth of *P. falciparum* and killing *An. funestus* s.s. larvae in vitro was demonstrated, especially for *C. citratus* essential oil. The accessibility of these materials, as well as the absence of reported ecotoxicity, make them promising models for the elaboration of new anti-malarial drugs and biological insecticides.
